# A Double-Negative Metamaterial-Inspired Mobile Wireless Antenna for Electromagnetic Absorption Reduction

**DOI:** 10.3390/ma8084817

**Published:** 2015-07-29

**Authors:** Touhidul Alam, Mohammad Rashed Iqbal Faruque, Mohammad Tariqul Islam

**Affiliations:** 1 Space Science Centre (ANGKASA), Universiti Kebangsaan Malaysia, UKM, Bangi 43600, Selangor, Malaysia; E-Mail: touhid13@siswa.ukm.edu.my; 2 Department of Electrical, Electronic and Systems Engineering, Universiti Kebangsaan Malaysia, UKM, Bangi 43600, Selangor, Malaysia; E-Mail: tariqul@ukm.edu.my

**Keywords:** antenna, double negative metamaterial, electromagnetic absorption, wireless communication

## Abstract

A double-negative metamaterial-inspired antenna is presented for mobile wireless applications. The antenna consists of a semi-circular radiating patch and a 3 × 4 hexagonal shaped metamaterial unit cell array in the ground plane. The antenna is fed with a 50 Ω microstrip feed line. The electric dimensions of the proposed antenna are 0.20λ × 0.26λ × 0.004λ, at the low-end frequency. The proposed antenna achieves a −10 dB impedance with a bandwidth of 2.29 GHz at the lower band and 1.28 GHz at the upper band and can operate for most of the mobile applications such as upper GSM bands, WiMAX, Bluetooth, and wireless local area network (WLAN) frequency bands. The focused novelties of the proposed antenna are its small size, multi-standard operating bands, and electromagnetic absorption reduction at all the operating frequencies using the double-negative metamaterial ground plane.

## 1. Introduction

Following the recent advent of artificial metamaterials, there has been much interest in microwave applications. Metamaterials are engineered materials that are usually formed by embedding periodic unit cells to produce exotic electromagnetic properties that are naturally unavailable, such as inverted Snell’s law or a negative refractive index. Some of these materials can have either negative permittivity or negative permeability at some frequencies, referred to as single negative (SNG) materials. If both negative permittivity (ε) and negative permeability (μ) are found at a certain frequency, the composite material exhibits a negative refractive index (η) property, and it is usually referred to as a double-negative (DNG), negative refractive index (NRI), or left-handed material (LHM). These unconventional properties of metamaterials are used in many current applications, such as microwave component design, antenna design, electromagnetic absorption reduction, contactless measurement, and invisibility cloaking [1,2,3,4].

Much study on the human health risk due to electromagnetic (EM) field radiation from wireless devices is in progress. Many short- and long-term effects of EM radiation on human health, such as disorders in sleep, cognitive function, heart rate, blood pressure, headaches, and brain tumors, are being studied by various health organizations like the World Health Organization (WHO). Now, several international organizations [5,6] have established guidelines for radio frequency exposure from wireless devices. The electromagnetic absorption limit recommended by the International Commission on Non-Ionizing Radiation Protection (ICNIRP) and IEEE C95.1:2005 guideline is 1.6 W/kg averaged over 1 gram of tissue volume in the shape of a cube and 2.0 W/kg average over any 10 grams of continuous tissue.

In recent years, extensive research efforts have been devoted to electromagnetic absorption reduction from mobile handset antennas. Different methods have been used to reduce EM absorption, such as embedding ferrite sheets [7,8], parasitic elements [9], artificial magnetic conductors, electromagnetic band gaps [10], and metamaterials [11,12,13]. In [14], the author presents the SAR reduction using metamaterial, but did not provide detailed information. Tay *et al.* proposed a reflector with a dipole to reduce the electromagnetic absorption in [15]. The drawback of this technique is the use of an additional reflector together with the main antenna, resulting in increased manufacturing cost and device dimensions. Kitra *et al.* investigated the EM absorption reduction upon the inclusion of ferrite in a material-loaded antenna and succeeded in reducing the EM absorption by 88% compared to conventional phones [8]. Though the ferrite material has special properties of permittivity and permeability to reduce EM absorption, it increases the manufacturing cost. In [9], Zhan *et al.* combined PIFA and a side-mounted inverted “F” antenna (IFA) for multifunctional applications as commercially needed and compared the SAR value with that of a conventional PIFA antenna. Although a reduction of 30% was achieved by combining a PIFA with a long IFA as the parasitic element, a large space is required to mount with its wireless devices. Sultan *et al.* proposed an EBG structure embedded antenna to reduce the maximum SAR [10]. In [11], Rashed *et al.* proposed a DNG metamaterial structure, which can be attached to the PCB to reduce the EM absorption. The major drawback of this technique is that the metamaterial structure needs additional space to mount with the PCB.

Antenna researchers are also extensively researching the minimization of the antenna size and cost, together with increasing the bandwidth to cover multiband. Chang *et al.* developed a Penta-band printed PIFA antenna for WLAN operation in a mobile phone [16] that can operate in two wide bands at approximately 900 MHz and 1900 MHz. In [17], Jie *et al.* presented a printed octaband monopole antenna for mobile phones sized at 15 × 26 mm^2^, which can operate in GSM850 (824–894 MHz), GSM900 (880–960 MHz), DCS (1710–1880 MHz), PCS (1850–1990 MHz), UMTS (1920–2170 MHz), and WiMAX (3400–3600 MHz). Chen *et al.* proposed a modified T-shaped planar antenna for wireless mobile applications that can operate in the DCS, UMTS, and lower and higher WLAN frequency bands [18]. The proposed antenna size was quite larger for mobile applications, which was 65 × 40 mm^2^. In [19], a crescent-shaped mobile wireless antenna was presented. The presented antenna can cover the frequency bands of 1.7 to 3.1 GHz, with antenna dimensions of 57 × 37.5 × 0.8 mm^3^. Sung *et al.* presented a modified L-shaped feed antenna that achieved an impedance bandwidth of 3.51 GHz (1.21–4.72 GHz) [20]. The antenna dimension was also larger than convenient for mounting on mobile devices.

In this paper, a metamaterial-loaded microstrip patch antenna is proposed for mobile wireless communication systems. The hexagonal metamaterial structure is embedded on the ground plane to reduce the maximum electromagnetic radiation of the proposed antenna. Moreover, the antenna performance has been investigated. This paper is structured as follows. Section 2 describes the structural design of the proposed antenna and unit cell array. Metamaterial characterization is included in Section 3. The proposed antenna performance is discussed in Section 4. The specific absorption rate analysis is discussed in Section 5, and Section 6 concludes the paper.

## 2. Design of the Proposed Antenna and Unit Cell

**Figure 1:**
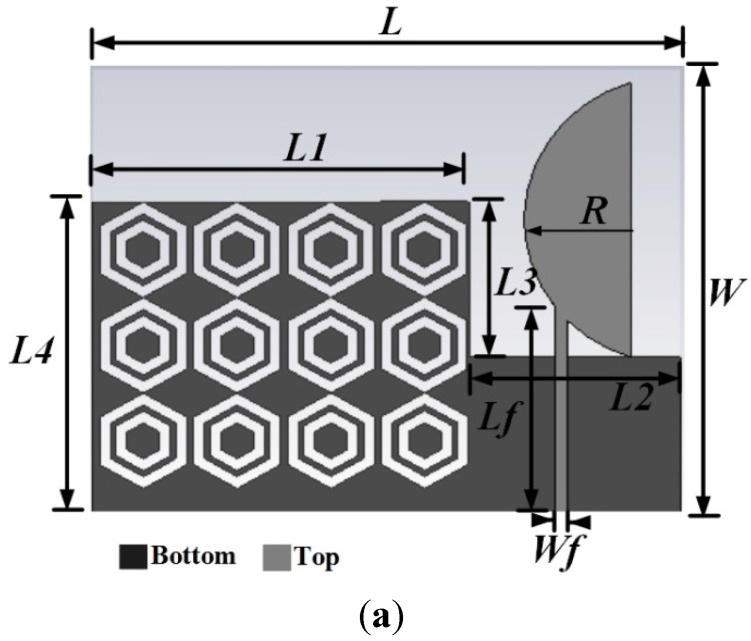

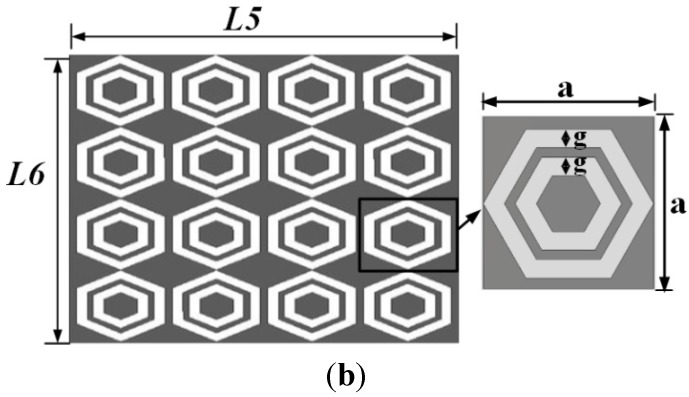
(**a**) Schematic diagram of the antenna; (**b**) Unit cell array and unit cell configuration.

The proposed metamaterial antenna and unit cell structure is presented in Figure 1. A hexagonal shaped metamaterial unit cell array is designed and fabricated on a 0.8 mm thick FR-4 substrate. The proposed antenna is also printed on a 0.8 mm thick FR-4 substrate of dimensions 45 × 35 mm^2^. The antenna is incorporated with a semi-circular patch and a hexagonal shaped metamaterial array in the ground plane. The semi-circular patch is printed on the top layer, and the metamaterial array is printed on the bottom layer of the substrate material. The antenna and unit cell design specifications are listed in Table 1.

**Table 1:** Antenna design and unit cell specifications.

Parameter Name	Value (mm)	Parameter Name	Value (mm)
L	45	L3	12
W	35	L4	13
Lf	17	L5	32
Wf	1.25	L6	28
L1	30	a	8
L2	15	g	0.8

## 3. DNG Metamaterial Characterization

**Figure 2:**
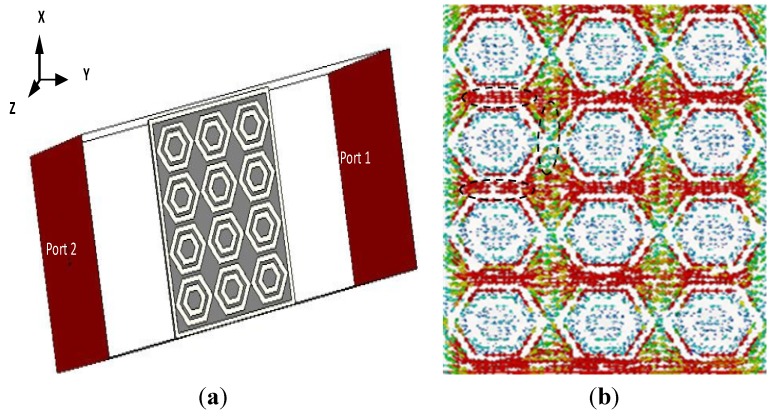
(**a**) Simulation arrangement of a unit cell array of metamaterial characteristics; (**b**) Surface current distribution at 1.97 GHz.

**Figure 3:**
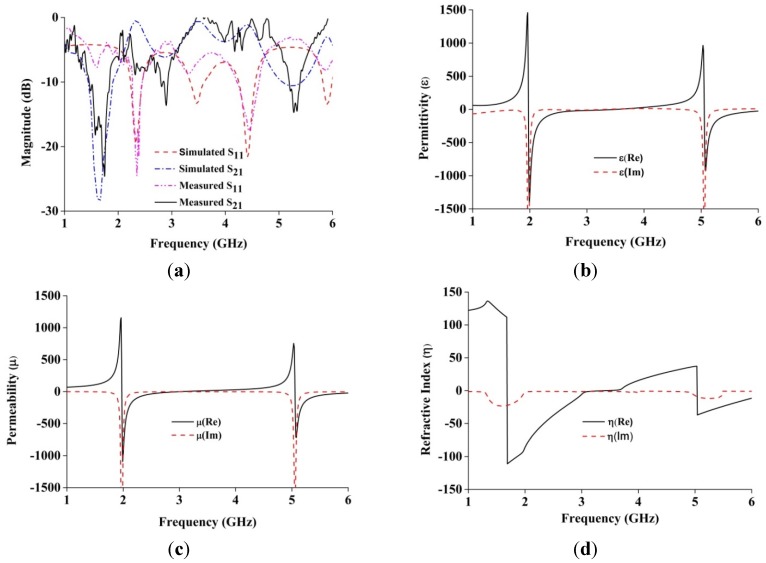
(**a**) Spectral response of the metamaterial structure; (**b**) Real and imaginary values of effective permittivity (ε) *vs.* frequency; (**c**) Real and imaginary values of effective permeability (µ) *vs.* frequency; (**d**) Real and imaginary values of refractive index (η) *vs.* frequency.

The metamaterial structure interacts with electromagnetic waves and shows some special properties. For characterizing the metamaterial, the array structure was positioned between two waveguide ports on the negative and positive x-axis and excited by a transverse electromagnetic (TEM) wave. The perfect electric conductor (PEC) boundary and the perfect magnetic conductor (PMC) boundary were defined along the y and z axes, respectively, as shown in Figure 2a. A frequency solver with a tetrahedral mesh was used for simulation. The normalized impedance was set to 50 Ω. The simulation was run in the frequency range of 1–6 GHz for both the metamaterial and antenna performance investigations. The constitutive parameters of the metamaterial were retrieved using scattering parameters, the method used in [21] and presented in Figure 3. The measured and simulated spectral analyses of the proposed metamaterial structure are illustrated in Figure 3a. It may be observed from Figure 3 that there are two resonance points at 1.963 GHz and 5.03 GHz where the DNG characteristics of the metamaterial have been found. It is shown from Figure 3b that the the retrieved negative permittivity regions of the the structure are found 1.97–3.03GHz and 5.0–6.0 GHz. Moreover, the retrieved negative permeability regions are 1.96–3.5 GHz and 5.05–6 GHz. Similarly, refractive index regions are obtained at 1.68–3.43 GHz and 5.04–6.0 GHz. Therefore, the metamaterial structure achieves double-negative medium of about 1.50 GHz at the lower band and about 0.95 GHz at the upper band. The magnetic resonance behavior can be assumed by observing simulated current distributions, as shown in Figure 2b, and comparing them with the existing behavior of the metamaterials [22,23]. It is shown from Figure 2b that the resonant electric current oscillates along the finite conductor. The parallel finite conductor can be considered as an LC resonant circuit. The inductance of the structure is formed by self- and mutual inductance of the conductors and capacitance is introduced between the gaps. Moreover, the periodic arrangement of the unit cells has an additional coupling between adjacent unit cells.

## 4. Antenna Performance Analysis

**Figure 4:**
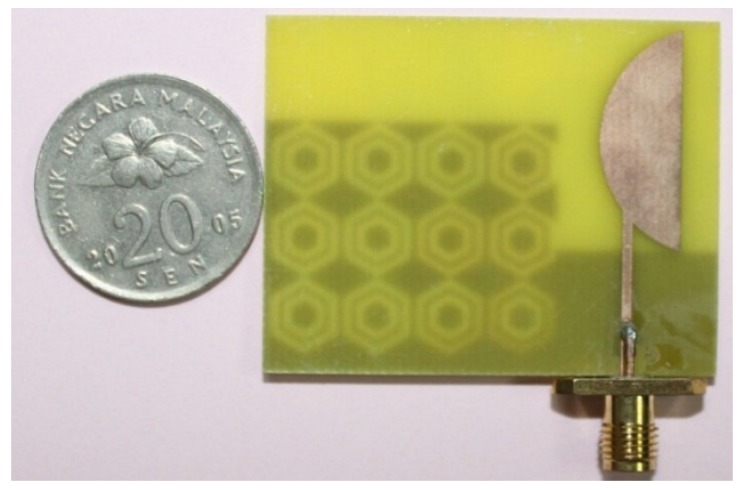
Proposed antenna fabricated prototype.

**Figure 5:**
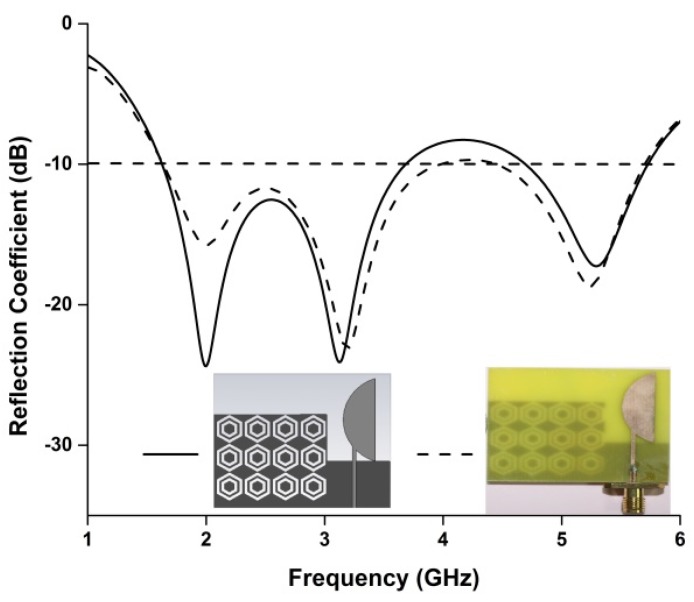
Simulated and measured reflection coefficients of the proposed antenna.

A prototype of the antenna has been fabricated using an LPKF Laser and Electronics machine and is shown in Figure 4. The reflection coefficient of the proposed antenna has been measured using a PNA network analyzer, presented in Figure 5. The proposed antenna achieved measured impedance bandwidths of 2.29 GHz (1.66–3.95 GHz) and 1.28 GHz (4.45–5.73 GHz), enabling it to operate in the frequency bands of GSM (1800, 1900, 2100), WiMAX (3.2–3.6 GHZ), Bluetooth (2.4 GHz), and WLAN (5.47–5.9 GHz). Although slight disagreement is found between the measured and simulated reflection coefficients, the two results are most likely identical. The main reasons for the disagreement between the two results are fabrication tolerance and deficient soldering effects of the SMA connector.

**Figure 6:**
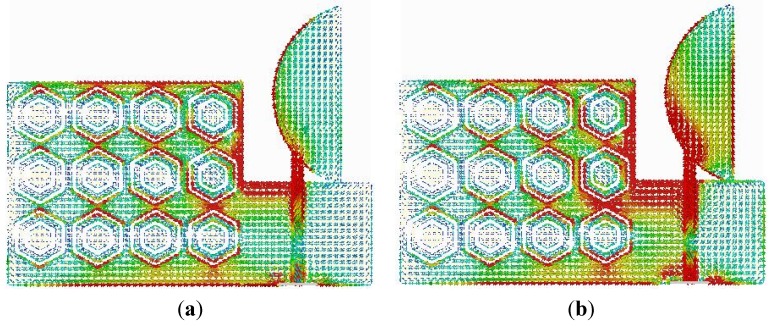
Surface current distribution of the proposed antenna at (**a**) 1.8 GHz; (**b**) 2.4 GHz.

To observe the physical phenomenon of the proposed antenna, the current distribution at different frequencies is analyzed. The surface current distribution is obtained from simulation software for different frequencies, as shown in Figure 6. A stronger surface current distribution is observed along the metamaterial ground plane and near the feed line.

**Figure 7:**
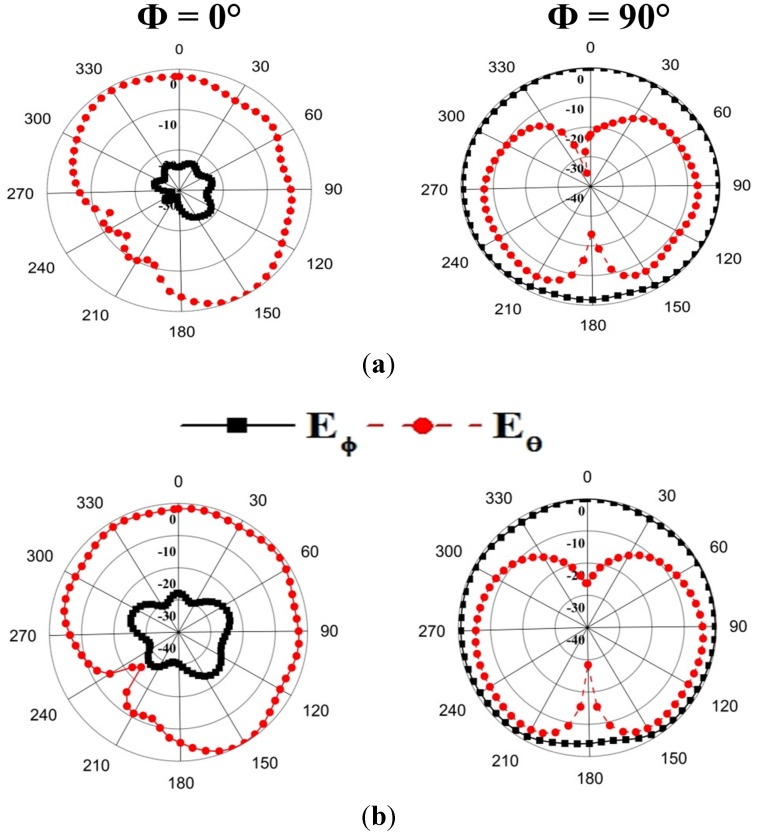
Measured radiation pattern of the proposed antenna. (**a**) 1.8 GHz; (**b**) 2.4 GHz.

The radiation pattern of the proposed antenna has been measured using the Satimo nearfield measurement system (Satimo Starlab). The measured radiation patterns at 1.8 and 2.4 GHz are demonstrated in Figure 7 where both Phi = 0° and Phi = 90° are included. It is seen from Figure 7a,b that the radiation patterns at Phi = 90° are nearly omnidirectional for Eϕ. According to the experimental result, it is seen that for the overall antenna volume, the proposed antenna with the compact size of 37 × 47 × 0.8 mm^3^ has an antenna size at least 33% less than [18], 18.5% less than [19], and 80% less than [20], and shows better antenna performances.

## 5. Electromagnetic Absorption Analysis

**Figure 8:**
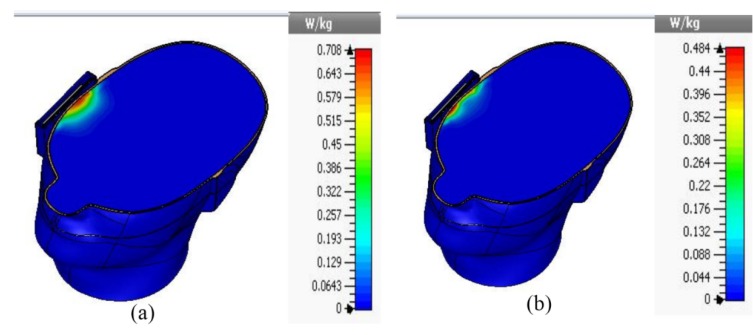
Simulated 1 g SAR values of the proposed antenna (**a**) at 1.8 GHz and (**b**) at 2.4 GHz.

The specific absorption rate of the proposed antenna has been studied using a commercially available ﬁnite-difference time-domain (FDTD) method-based CST microwave studio. The simulation arrangement was set up according to IEEE and Federal Communications Commission (FCC) guidelines. The input power was set to 500 mW, and the distance between the head phantom and the mobile phone was approximately 2 mm. The SAM head phantom consists of head equivalent liquid (ɛ_r_ = 40, σ = 1.4) and shell (ɛ_r_ = 5, tangent delta = 0.05). The simulated 1 g SAR at 1.8 GHz and 2.4 GHz has been analyzed and is presented in Figure 8. It is shown in Figure 8 that the metamaterial antenna shows 1 g SAR values at 1.8 GHz and 2.4 GHz of 0.708 W/Kg and 0.484 W/Kg, respectively. The simulated SAR values of the proposed metamaterial-loaded antenna are much lower than the standard safety guidelines. Here, the metamaterial structure plays the most important role in reducing the SAR values. The metamaterial structure has high electromagnetic surface currents and acts as a perfect magnetic conductor (PMC) in a specified frequency range. Moreover, the stop band characteristics of the metamaterial structure can control the radiation characteristics of the antenna. These characteristics of the metamaterial can reduce the undesirable EM waves that travel to the human head without degrading the antenna performance.

**Figure 9:**
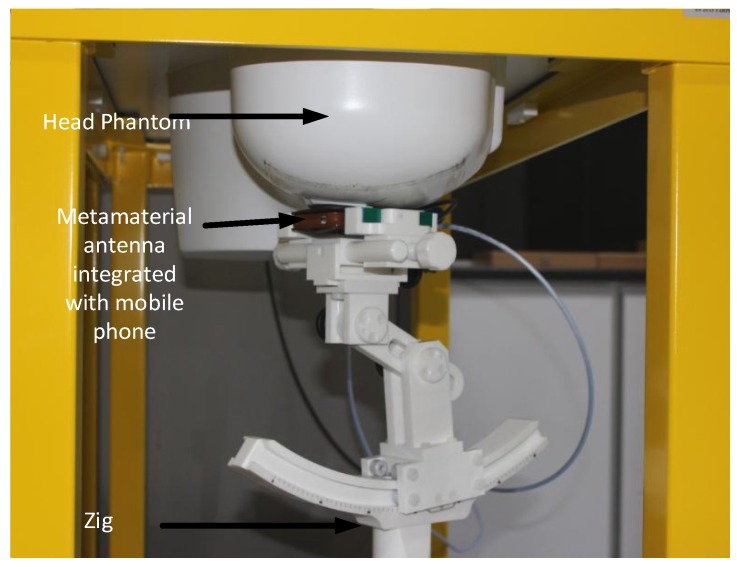
SAR measurement in the Satimo SAR measurement lab.

**Figure 10:**
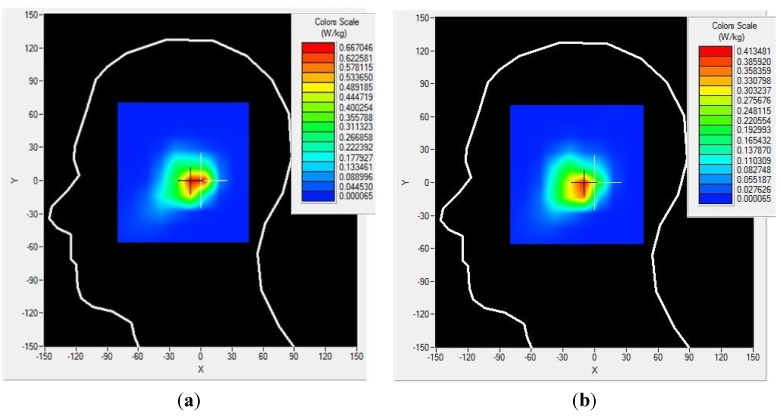
1 g SAR measurements of the proposed antenna at (**a**) 1.8 GHz and (**b**) 2.4 GHz.

The SAR values of the proposed antenna have been measured using the Satimo COMOSAR measurement system. The system consists of a robot to move the field probe, head phantom, and test zig, as shown in Figure 9. The field probe is connected to the system computer. The head phantom is filled with liquid, which maintains the equivalent dielectric properties of the human head. The metamaterial antenna-loaded mobile phone was placed in a test zig and connected with an input power supply set at 27 dBm (500 mW). The distance between the head phantom and the mobile phone was approximately 6 mm. The measurement was performed at 1.8 and 2.4 GHz. The measured 1 g SAR value of the proposed antenna is shown in Figure 10, and the simulated and measured results are listed in Table 2. It is seen from Table 2 that the proposed antenna has succeeded in a large-scale reduction of SAR values as compared to reported antennas.

**Table 2:** SAR values of the proposed antenna.

Type	1g SAR (W/Kg)			
	Condition	Frequency (GHz)	SAR values (W/Kg)	S_11_ (dB)
**Metamaterial antenna**	simulated	1.8	0.708	−16
	measured	1.8	0.667	−14.8
	simulated	2.4	0.484	−13.5
	measured	2.4	0.413	−14.2

**Figure 11:**
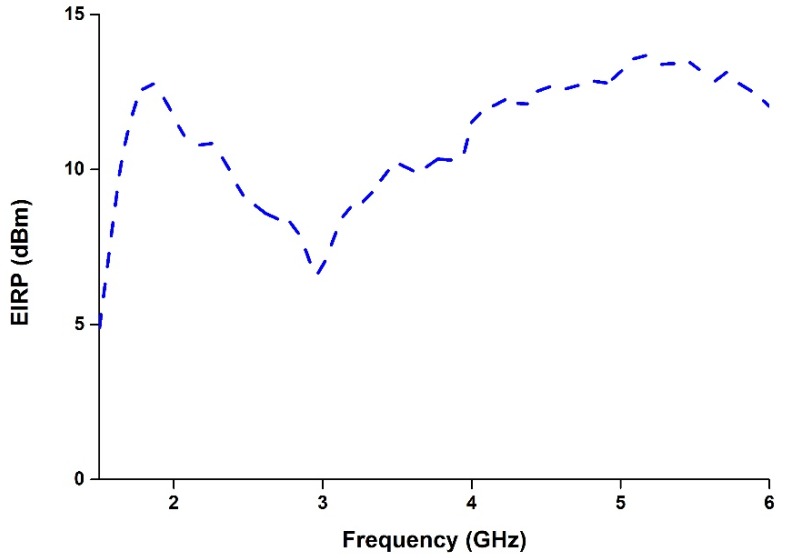
Equivalent isotropic radiated power (EIRP) of the proposed antenna.

The equivalent isotropic radiated power (EIRP) is a very important criterion that all wireless equipment and devices must satisfy to minimize the exposure of human beings to electromagnetic fields. The EIRP is related to the power transmitted (*Pt*), cable losses (*Lc*), and the antenna gain (*Ga*), and its expression is presented in Equation 1. The EIRP of the proposed antenna has been calculated and is presented in Figure 11. It is seen from Figure 11 that the antenna satisfies the EIRP limit for wireless applications.

(1) ETRP [dBm]=Pt [dBm]−Lc [dB]+Ga [dBi]

## 6. Conclusions

A low-profile metamaterial antenna has been presented for low electromagnetic absorption mobile applications. The proposed antenna with a metamaterial structure was found to reduce the peak SAR values without degrading the antenna performance. The measured 1 g SAR values of the proposed antenna were 0.667 W/kg and 0.413 W/kg at 1.8 GHz and 2.4 GHz, respectively, which are 58.31% and 74.19% lower than the standard safety guidelines. Therefore, the human body can be sheltered from the hazardous effects of the electromagnetic radiation using the proposed antenna.
